# Particulate Matter 10 (PM_10_) Is Associated with Epistaxis in Children and Adults

**DOI:** 10.3390/ijerph18094809

**Published:** 2021-04-30

**Authors:** Kyungsoo Kim, Il-Youp Kwak, Hyunjin Min

**Affiliations:** 1 Department of Otorhinolaryngology-Head and Neck Surgery, Chung-Ang University College of Medicine, 224-1 Heukseok-dong, Dongjak-gu, Seoul 156-755, Korea; entkks@cau.ac.kr; 2 Department of Applied Statistics, Chung-Ang University, 224-1 Heukseok-dong, Dongjak-gu, Seoul 156-755, Korea; ikwak2@cau.ac.kr; 3 Biomedical Research Institute, Chung-Ang University Hospital, Seoul 156-755, Korea

**Keywords:** epistaxis, humidity, meteorological concepts, particulate matter, temperature

## Abstract

The impact of atmospheric concentration of particulate matter ≤10 μm in diameter (PM_10_) continues to attract research attention. This study aimed to evaluate the effects of meteorological factors, including PM_10_ concentration, on epistaxis presentation in children and adults. We reviewed the data from 1557 days and 2273 cases of epistaxis between January 2015 and December 2019. Eligible patients were stratified by age into the children (age ≤17 years) and adult groups. The main outcome was the incidence and cumulative number of epistaxis presentations in hospital per day and month. Meteorological factors and PM_10_ concentration data were obtained from the Korea Meteorological Administration. Several meteorological factors were associated with epistaxis presentation in hospital; however, these associations differed between children and adults. Only PM_10_ concentration was consistently associated with daily epistaxis presentation in hospital among both children and adults. Additionally, PM_10_ concentration was associated with the daily cumulative number of epistaxis presentations in hospital in children and adults. Furthermore, the monthly mean PM_10_ concentration was significantly associated with the total number of epistaxis presentations in the corresponding month. PM_10_ concentration should be regarded as an important environmental factor that may affect epistaxis in both children and adults.

## 1. Introduction

Epistaxis is one of the most common clinical presentations in the general population. Approximately 50% of children experience at least one episode of epistaxis [1], and 60% of the general population experiences epistaxis at least once in their lifetime [2]. Since cases of epistaxis are commonly encountered in the otorhinolaryngology department of hospitals, multiple studies have investigated factors associated with such presentations. Previous reports have suggested a correlation between epistaxis presentation at the hospital and meteorological factors [2,3,4]. Factors such as air temperature, relative humidity, and wind speed, in particular, have been associated with the number of epistaxis presentations. Moreover, recent studies have reported significant correlations between air pollutants and meteorological factors and the incidence of epistaxis [5,6]. However, only a few studies have evaluated the differences in the impact of meteorological factors, including air pollutants, on the incidence of epistaxis in both children and adults.

Epistaxis in adults is considered to be different from that in children. Most childhood epistaxis is spontaneous and self-limiting in nature, and originates from the Little’s area, which is located at the anterior region of the nasal septum, where several terminal vessels anastomose with each other, forming a plexus [7,8]. However, posterior epistaxis is more common in adults than in children, and usage of anticoagulants and presence of hypertension may be associated with epistaxis in adults [9]. Therefore, the association between meteorological factors/or air pollutants and epistaxis may be different in adults and children.

Exposure to ambient particulate matter (PM) and its effect on human health is one of the major challenges in South Korea, where the PM concentration is higher than that in North America, Western Europe, and Japan [10]. PM refers to the complex mixture of organic and inorganic solid particles and liquid droplets found in the atmosphere [11]. It is associated with various adverse health conditions, such as respiratory and cardiovascular dysfunction, and those requiring hospitalization and emergency room visits [12,13]. Particle size has been shown to be directly associated with the risk of adverse health outcomes, and PM ≤ 10 μm in diameter (PM_10_) tends to settle in the upper rather than the lower airway [14]. Moreover, it has been associated with respiratory symptoms, such as cough, sore throat, and sputum production [15]. To date, no study has investigated the differential effects of PM_10_ on epistaxis incidence in both children and adults in countries with high atmospheric concentrations of PM_10_. In this study, we aimed to evaluate the effects of meteorological factors, including atmospheric PM_10_ concentration, on epistaxis presentation at hospitals in both children and adults.

## 2. Materials and Methods

This study was approved by the Institutional Review Board of Chung-Ang University College of Medicine (2002-013-19303). The requirement for informed consent was waived by the institutional review board due to the retrospective study design.

### 2.1. Study Design

We retrospectively reviewed the medical records of patients who presented with spontaneous epistaxis at the Chung-Ang University Hospital between January 2015 and July 2019. Patients who presented with secondary epistaxis due to bleeding disorders, a foreign body lodged in the nose, or a history of trauma and/or nasal surgery were excluded from the study. Eligible patients were stratified by age into a children group (age ≤17 years) [16] and an adult group.

The presence of hospital epistaxis presentation per day and the cumulative number of epistaxis presentations per day were considered as outcome variables in the statistical analysis. Meteorological data and data on PM10 concentration are considered as exposure variables, and they were obtained from the Korea Meteorological Administration (https://web.kma.go.kr/eng/index.jsp) (accessed on 1 September 2019). Data on daily air pressure (hPa), wind speed (m/s), air temperature (°C), relative humidity (%), sunshine duration (h), amount of solar radiation (MJ/m^2^), number of clouds (cloud cover) (1/10), and PM10 concentration (μg/m^3^) were analyzed for the period between 1 January 2015 and 31 December 2019 (data summary in Supplementary Table S1).

### 2.2. Statistical Analysis

Exposure variables and the outcome measurements were obtained from the same city to reduce the spatial variations. Exposure variables whose *p*-value was < 0.05 in univariate analysis were selected for multivariate analysis.

Univariate and multivariate logistic regression analyses were performed to identify the meteorological factors, including PM_10_ concentration, associated with the presence of epistaxis presentations in hospital per day. We randomly divided the entire dataset into training and evaluation data according to an 8:2 ratio. We trained separate multivariate logistic regression models for the children and adult groups. We performed the stepwise selection procedure according to the Akaike information criterion (AIC) to select the exposure variables in our multivariate logistic regression model using the training data. The mean temperature, mean wind speed, sunshine duration, and PM_10_ concentration were the variables selected for the children’s model, and the minimum temperature, mean relative humidity, number of clouds, and PM_10_ concentration were the variables selected for the adults’ model.

In the next step, we tried to further identify exposure variables that affect increased number of epistaxis presentations per day. We used three ordinal categories (whether the number of patients presenting with epistaxis per day was “0”, “1”, or “≥2”) as our outcome variables of the ordinal regression. The model also passed the Lipsitz test (goodness-of-fit test for ordinal regression). An ordinal logistic regression analysis was used to determine the meteorological factors, including PM_10_ concentration, associated with the cumulative number of epistaxis presentations per day. For the multivariate ordinal logistic regression analysis, we randomly divided the entire dataset into training and evaluation data in an 8:2 ratio. We trained separate multivariate logistic regression models for the children and adult groups. We performed the stepwise selection procedure according to the AIC to select the exposure variables in our multivariate ordinal logistic regression model using the training data. The mean temperature, mean wind speed, sunshine duration, and PM_10_ concentration were the variables selected for the children’s model, and the maximum temperature, maximum wind speed, mean relative humidity, and PM_10_ concentration were the variables selected for the adults’ model.

Values of the are presented as means (standard deviations (SDs) and ranges). Pearson correlation coefficients were used to evaluate the relationship between two continuous variables. A *p*-value of <0.05 was considered indicative of a statistically significant finding. All analyses were performed using R and SPSS for Windows version 20.0 (IBM Corp., Armonk, NY, USA)

## 3. Results

### 3.1. Characteristics of the Enrolled Subjects

We reviewed the medical records from 1704 days. A total of 2249 cases were approached, and 692 cases were excluded because they were thought to be secondary epistaxis cases; finally, 1557 cases of epistaxis were identified. The mean age of the patients was 44.94 (SD: 25; range: 1–94) years (Table 1). Among them, 20.7% (323/1557) were children, and 79.3% (1234/1557) were adults. The mean age of the children was 7.59 (SD: 4.41; range: 1–17) years, and that of the adults was 60.00 (SD 18.85; range: 18–94) years (Table 1). Meteorological factors such as cloud cover, humidity, temperature, air pressure, solar radiation, sunshine duration, wind speed, and PM_10_ concentration were evaluated as exposure variables (Supplementary Table S1).

### 3.2. Monthly Distribution of Meteorological Factors and PM_10_ Concentration

**Figure 1:**
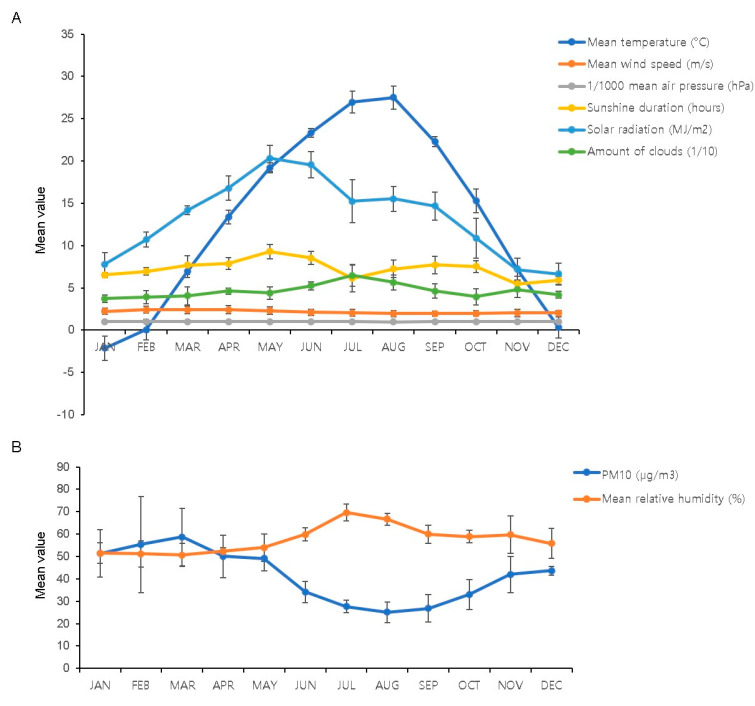
Monthly distribution of the monthly average of the meteorological factors and PM_10_ concentration from 2015 to 2019. (**A**) Monthly mean value of temperature, wind speed, air pressure (1/1000 scale), and sunshine duration. (**B**) Monthly mean value of relative humidity and PM_10_ concentration. PM_10_: particulate matter <10 μm in diameter.

**Figure 2:**
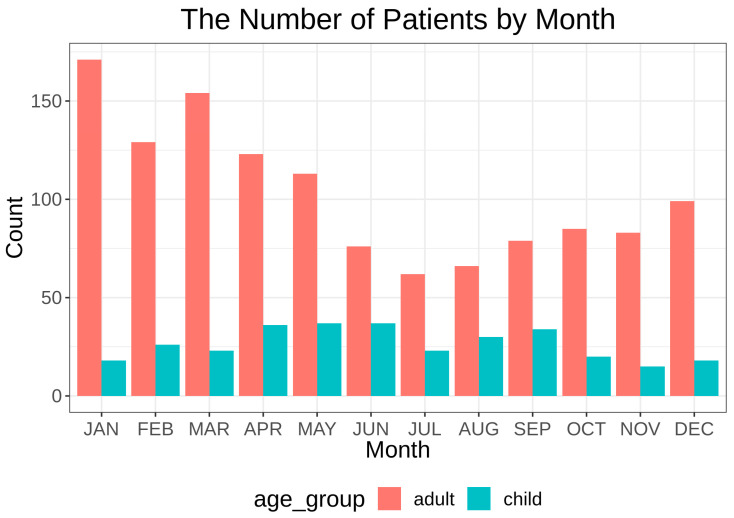
Monthly distribution of the number of epistaxis cases among children and adults from 2015 to 2019.

The coldest month of the year was January, with a mean daily temperature of −2.14 °C, followed by February (0.05 °C) and December (0.33 °C) (Figure 1A). The hottest month was August, with a mean daily temperature of 27.49 °C, followed by July (26.95 °C) and June (23.32 °C). The lowest monthly mean relative humidity was observed in March (50.67%), followed by February (51.13%) and January (51.50%) (Figure 1B). The monthly mean relative humidity was highest in July (69.61%) and August (66.61%). The highest mean wind speed was evident in February (2.41 m/s), followed by March (2.38 m/s) (Figure 1A). The highest mean air pressure was observed in December (10,150 hPa) (Figure 1A). The month with the highest duration of sunshine and greatest amount of solar radiation was May (9.28 h, 20.31 MJ/m^2^) (Figure 1A). The number of clouds was highest in July (6.46 [1/10]) and lowest in January (3.74 [1/10]) (Figure 1A). The mean concentration of PM_10_ was highest in March (58.66 μg/m^3^), followed by February (55.34 μg/m^3^) and January (51.38 μg/m^3^) (Figure 1B). When we reviewed the number of epistaxis cases, we observed that the children and adults showed different patterns. The adult group had the highest number of patients during the winter months, whereas the children group had the highest number of cases during the summer months (Figure 2 and Supplementary Figure S1). We further classified the age of the adult group into three subdivisions, “18–39 years”, “40–69 years”, and “>70 years”. All age groups among the adults showed a similar pattern of lesser number of epistaxis cases during the summer months (Supplementary Figure S2).

### 3.3. Factors Associated with the Incidence of Epistaxis Presentation in Hospital per Day among Children and Adults

The associations between meteorological factors, including PM_10_ concentration, and epistaxis presentation in hospital were evaluated using multivariate logistic regression analysis (Table 2). Among children, mean temperature, mean wind speed, sunshine duration, and PM_10_ concentration were positively associated with the incidence of epistaxis presentation in hospital. Among adults, the number of clouds and PM_10_ concentration were positively associated with the incidence of epistaxis presentation in hospital, whereas minimum temperature and mean relative humidity were negatively associated with the incidence of epistaxis presentation in hospital (Table 2).

To check the linearity assumption, we visualized the relationship between the logit value of the predicted probability and the statistically significant exposure variables (Supplementary Figure S3). We observed a particularly strong linear relationship with temperature and PM_10_ concentration using predicted logit probability (Supplementary Figure S3).

### 3.4. Factors Associated with the Number of Epistaxis Presentations in Hospital per Day among Children and Adults

We evaluated the impact of meteorological factors, including PM_10_ concentration, on the cumulative number of epistaxis presentations in hospital per day using ordinal regression analysis (Table 3). Increased mean temperature, mean wind speed, sunshine duration, and PM_10_ concentration were associated with an increase in the daily number of epistaxis cases among children. In adults, increased PM_10_ concentration was associated with an increase in the daily number of epistaxis cases, whereas increased maximum temperature, maximum wind speed, and mean relative humidity were associated with fewer cases.

### 3.5. Additive Effect of PM_10_ Concentration on Epistaxis Presentation in Hospital

**Figure 3:**
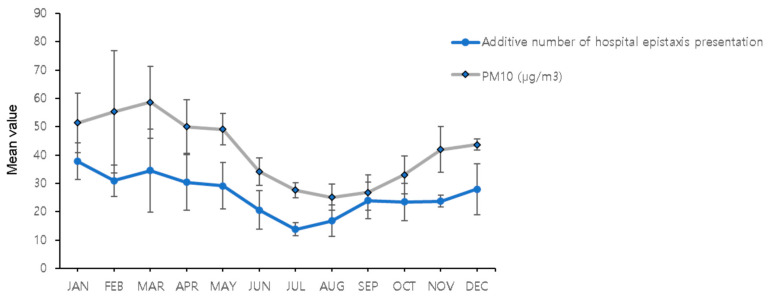
Relationship between monthly mean PM_10_ concentration and total number of epistaxis presentations in hospital within the corresponding month. PM_10_: particulate matter <10 μm in diameter.

To evaluate the additive effects of PM_10_ concentration, we calculated the mean PM_10_ concentration per month and examined those against the total number of epistaxis presentations in hospital within the same month (Figure 3). We observed that the monthly mean PM_10_ concentration was positively (Pearson’s coefficient = 0.884, *p* < 0.001) associated with the total number of epistaxis presentations in hospital within that same month.

## 4. Discussion

This study evaluated the effects of meteorological factors, including PM_10_ concentration, on children and adults presenting with epistaxis at our hospital. PM_10_ concentration was the only factor that consistently correlated with epistaxis presentation in hospital among both children and adults. Furthermore, PM_10_ concentration was associated with the cumulative number of epistaxis presentations in hospital among both children and adults. These findings indicate PM_10_ concentration may be a risk factor for epistaxis in both children and adults.

Multiple studies have investigated the relationships between meteorological factors and epistaxis. Although the results have been inconsistent, most of the evidence shows humidity to be closely associated with epistaxis. This association is plausible, given that low humidity may cause dryness of the nasal mucosa, which may lead to nasal bleeding [11]. Meanwhile, findings regarding the impact of temperature have been heterogenous, including some studies reporting a positive correlation between temperature and epistaxis in children [17], and other studies reporting the opposite association [18]. Results for other meteorological factors, such as air pressure, sunshine duration, and wind speed have also been inconsistent. For example, a previous study reported that while air pressure and sunshine duration were negatively and positively correlated with epistaxis incidence, respectively, the mechanisms underlying these relationships were unclear [2]. These results suggest that the effect of each meteorological factor on epistaxis is likely to be mediated by other factors, for example, geographic or climatic conditions.

PM_10_ concentration is associated with skin diseases, respiratory diseases, cardiovascular diseases, and mental health problems [19,20,21,22]. However, the effect of PM_10_ concentrations on epistaxis has rarely been studied. In this study, PM_10_ concentration was associated with the presence of and the daily number of epistaxis presentations in hospital in both children and adults. Furthermore, the additive effects of PM_10_ concentrations were analyzed by calculating the average monthly PM_10_ concentration and examining it against the total number of epistaxis cases in the corresponding month (Figure 3). Several studies have proposed a possible mechanism through which PM_10_ may induce epistaxis. Calderon et al. [23] observed that the nasal mucosa in children who were chronically exposed to air pollutants, such as PM_10_, showed basal cell hyperplasia, a decreased number of ciliated and goblet cells, neutrophilic epithelial infiltrates, squamous metaplasia, and mild dysplasia. These direct effects on the nasal mucosa may result in epistaxis. As PM_10_ is a mixture of constituents from multiple sources, including organic carbon, inorganic aerosols, and metal compounds, it can stimulate the release of inflammatory cytokines, making the nasal mucosa vulnerable to epistaxis [23]. Long-term exposure to PM_10_ is associated with an increase in blood pressure [24]. Further research is required to elucidate the direct and indirect mechanisms underlying the effects of PM_10_ on epistaxis. In contrast to our findings, a previous study reported a negative correlation between epistaxis occurrence and PM_10_ concentration in children [11]. However, the PM_10_ concentration was 10 times higher in our study than in the previous study. This discrepancy suggests that the effect of PM_10_ at various concentrations should be investigated further.

The mechanisms of epistaxis may be different in adults and children. A previous study reported that factors, such as chronic sinusitis, alcohol intake, and smoking, increase the risk of epistaxis in adults [11]. In children, epistaxis is seldom serious and is usually caused by digital trauma; it is largely self-limiting [25]. As children regularly attend school and engage in outdoor activities, environmental factors, such as weather and air conditions, have a greater influence on their health than on the health of adults [12]. Interestingly, regarding the association of meteorological factors with epistaxis between children and adults (Table 2 and Table 3), air temperature was positively associated with epistaxis in children and showed a negative association in adults. Further, wind speed was positively associated with the cumulative number of epistaxis presentation in hospital in children, and was negatively associated in adults. The only factor that showed consistent effects with epistaxis presentation in both children and adults was PM_10_ concentration. These findings indicate that meteorological factors may have different effects on children and adults; however, PM_10_ concentration may be an environmental risk factor of similar relevance among both children and adults.

Our study has several limitations. First, this was a retrospective study, and the patients did not have detailed clinical records. Therefore, the effects of other factors, such as allergic rhinitis, acute respiratory infections, and hypertension, were not evaluated. Additionally, the impact of confounders, such as rainfall and traffic, which may lead to patients being diverted from the hospital, was not evaluated. Moreover, misinformation induced by the retrospective chart review cannot be excluded. Second, although the effects of meteorological factors or PM_10_ concentration may persist for several days, we only considered the effects of these factors on a specific day and were unable to evaluate the lag effect. To compensate for this limitation, we calculated the monthly mean values of PM_10_ concentration and evaluated their correlations with the total number of epistaxis cases within that same month. An additional time series analysis in a future study may reveal the lag effects. Finally, this was a single-center study; hence, our findings may not be generalizable to other regions with different geographic and climatic characteristics.

## 5. Conclusions

In conclusion, meteorological factors showed different effects on epistaxis presentation in hospital in children and adults. Only PM_10_ concentration showed consistent effects on epistaxis presentation in hospital, and the number of epistaxis presentations in both children and adults. The physiological mechanisms underlying these results warrant further investigation.

## Figures and Tables

**Table 1:** Characteristics of the study participants.

	N
No. of days	1704
No. of epistaxis cases	1557
Age (years)	
Total	
Mean (SD)	44.94 (25.34)
Range	1–94
Children to adults	323:1234
Children	
Mean (SD)	7.59 (4.41)
Range	1–17
Adults	
Mean (SD)	60.00 (18.85)
Range	18–94

SD: standard deviation.

**Table 2:** Results of multivariate logistic regression analyses of meteorological factors, including PM_10_ concentration, and incidence of epistaxis presentation in hospital per day among children and adults.

Parameter	Univariate		Multivariate	
	Odds Ratio (95% CI)	*p*-Value	Odds Ratio (95% CI)	*p*-Value
**Children**				
Mean temperature (°C)	1.107 (1.005–1.030)	<0.001	1.025 (1.010–1.041)	<0.001
Minimum temperature (°C)	1.013 (1.001–1.026)	<0.001		
Maximum temperature (°C)	1.020 (1.007–1.032)	<0.001		
Mean air pressure (hPa)	0.979 (0.963–0.995)	0.013		
Maximum wind speed (m/s)	1.062 (0.991–1.137)	<0.001		
Mean wind speed (m/s)	1.364 (1.160–1.601)	<0.001	1.385 (1.143–1.674)	<0.001
Minimum relative humidity (%)	0.991 (0.982–1.000)	<0.001		
Mean relative humidity (%)	0.991 (0.982–1.000)	<0.001		
Sunshine duration (h)	1.083 (1.045–1.123)	<0.001	1.064 (1.022–1.108)	0.003
Solar radiation amount (MJ/m^2^)	1.045 (1.025–1.066)	<0.001		
Number of clouds (1/10)	0.943 (0.902–0.985)	<0.001		
PM_10_ (μg/m^3^)	1.004 (1.000–1.008)	<0.001	1.006 (1.001–1.011)	0.040
**Adults**				
Mean temperature (°C)	0.967 (0.958–0.976)	<0.001		
Minimum temperature (°C)	0.965 (0.956–0.974)	<0.001	0.970 (0.959–0.982)	<0.001
Maximum temperature (°C)	0.969 (0.960–0.978)	<0.001		
Mean air pressure (hPa)	1.043 (1.030–1.057)	<0.001		
Maximum wind speed (m/s)	0.989 (0.937–1.044)	0.684		
Mean wind speed (m/s)	1.076 (0.945–1.225)	0.271		
Minimum relative humidity (%)	0.987 (0.980–0.993)	<0.001		
Mean relative humidity (%)	0.986 (0.979–0.993)	<0.001	0.987 (0.977–0.997)	0.013
Sunshine duration (h)	0.987	0.329		
Solar radiation amount (MJ/m^2^)	0.978	0.003		
Number of clouds (1/10)	0.990 (0.958–1.023)	0.554	1.085 (1.035–1.139)	<0.001
PM_10_ (μg/m^3^)	1.013 (1.008–1.018)	<0.001	1.008 (1.003–1.014)	0.003

CI: confidence interval; PM_10_: particulate matter <10 μm in diameter.

**Table 3:** Results of ordinal logistic regression analysis of the meteorological factors, including PM_10_ concentration, and cumulative number of epistaxis presentations in hospital per day between children and adults.

Parameter	Univariate		Multivariate	
	Odds Ratio (95% CI)	*p*-Value	Odds Ratio (95% CI)	*p*-Value
**Children**				
Mean temperature (°C)	1.018 (1.005–1.030)	0.005	1.005 (1.010–1.041)	<0.001
Minimum temperature (°C)	1.014 (1.002–1.026)	0.025		
Maximum temperature (°C)	1.020 (1.008–1.033)	0.001		
Mean air pressure (hPa)	0.978 (0.978–0.978)	<0.001		
Maximum wind speed (m/s)	1.061 (0.990–1.135)	0.088		
Mean wind speed (m/s)	1.357 (1.155–1.591)	<0.001	1.385 (1.143–1.672)	<0.001
Minimum relative humidity (%)	0.991 (0.982–1.000)	0.058		
Mean relative humidity (%)	0.991 (0.982–1.000)	0.061		
Sunshine duration (h)	1.081 (1.044–1.122)	<0.001	1.063 (1.022–1.107)	0.003
Solar radiation amount (MJ/m^2^)	1.044 (1.025–1.066)	<0.001		
Number of clouds (1/10)	0.944 (0.904–0.986)	<0.001		
PM_10_ (μg/m^3^)	1.004 (1.000–1.008)	0.058	1.005 (1.001–1.010)	0.019
**Adults**				
Mean temperature (°C)	0.965 (0.956–0.973)	<0.001		
Minimum temperature (°C)	0.963 (0.955–0.971)	<0.001		
Maximum temperature (°C)	0.967 (0.959–0.976)	<0.001	0.970(0.960–0.980)	<0.001
Mean air pressure (hPa)	1.045 (1.0453–1.0455)	<0.001		
Maximum wind speed (m/s)	0.973 (0.925–1.023)	0.289	0.915(0.862–0.971)	0.003
Mean wind speed (m/s)	1.068 (0.955–1.207)	0.295	
Minimum relative humidity (%)	0.985 (0.978–0.991)	<0.001		
Mean relative humidity (%)	0.984 (0.977–0.990)	<0.001	0.991(0.984–0.999)	0.031
Sunshine duration (h)	0.993 (0.970–1.017)	0.563		
Solar radiation amount (MJ/m^2^)	0.977 (0.963–0.991)	0.001		
Number of clouds (1/10)	0.981 (0.951–1.012)	0.222		
PM_10_ (μg/m^3^)	1.010 (1.007–1.014)	<0.001	1.008(1.004–1.013)	<0.001

CI: confidence interval; PM_10_: particulate matter <10 μm in diameter.

## Data Availability

The data presented in this study are available on request from the corresponding author.

## Supplementary Materials

The following are available online at https://www.mdpi.com/article/10.3390/ijerph18094809/s1, Figure S1: The number of epistaxis presentation by month and year for the age group of child and adult. The adult group has the highest number of patients in winter periods, on the other hand the child group has its pick in summer periods, Figure S2: The number of epistaxis presentation by month of the adults group during 2015–2019. We further divided the age of the adult group in three components, ‘18–39,’ ‘40–69,’ and ‘over 70.’ All age groups in adults show a similar pattern of having smaller numbers of patients in summer periods, Figure S3: To check the linearity assumption, we visualized the relationship between logit value of the predicted probability and statistically significant exposure variables. Especially for temperature and pm10, we found a strong linear relationship with predicted logit probability, Table S1: Details of exposure variables.
