# Supplementary material for: Particulate Matter 10 (PM10) Is Associated with Epistaxis in Children and Adults
Source: Int J Environ Res Public Health. 2021 Apr 30;18(9):4809. doi: 10.3390/ijerph18094809 (PMC8124263; doi:10.3390/ijerph18094809)
Supplement: Supplementary file 1 [file ijerph-18-04809-s001.zip › Supplementary figure 1.pdf]

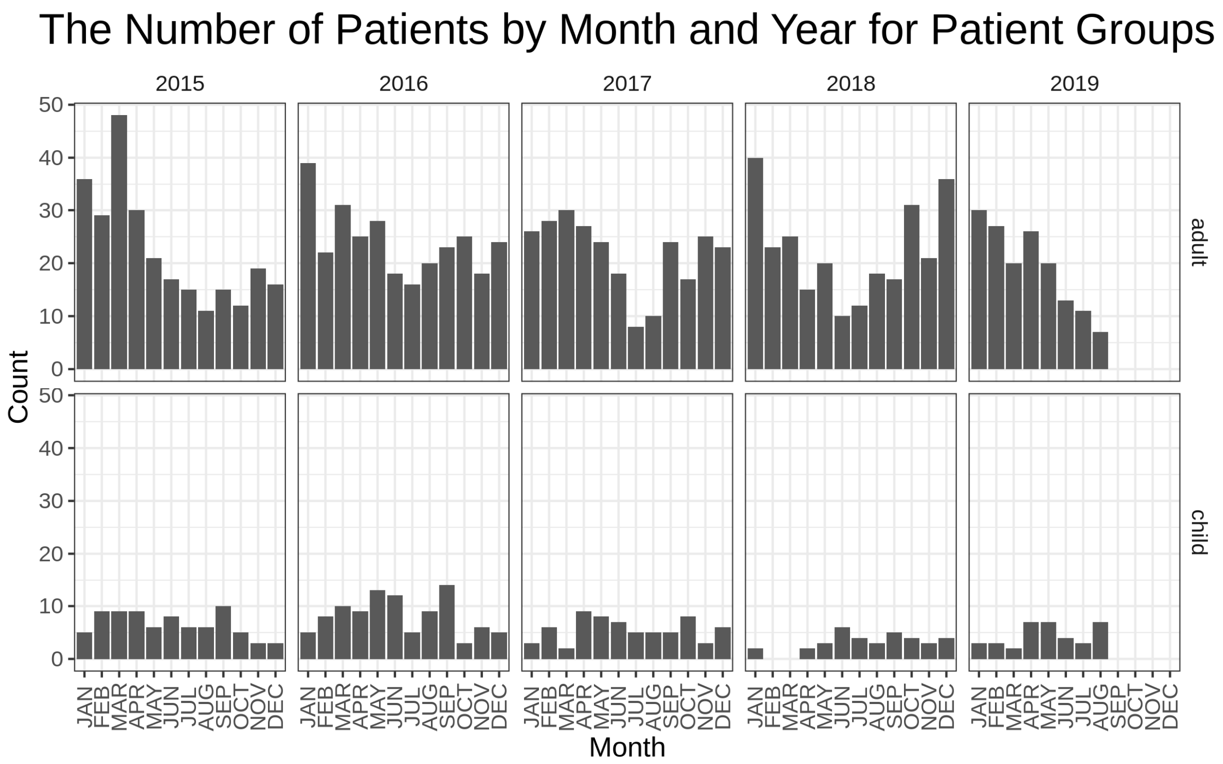

Supplementary figure 1. The number of epistaxis presentation by month and year for the age group of child and adult. The adult group has the highest number of patients in winter periods, on the other hand the child group has its pick in summer periods.
