# Supplementary material for: Particulate Matter 10 (PM10) Is Associated with Epistaxis in Children and Adults
Source: Int J Environ Res Public Health. 2021 Apr 30;18(9):4809. doi: 10.3390/ijerph18094809 (PMC8124263; doi:10.3390/ijerph18094809)
Supplement: Supplementary file 1 [file ijerph-18-04809-s001.zip › Supplementary figure 2.pdf]

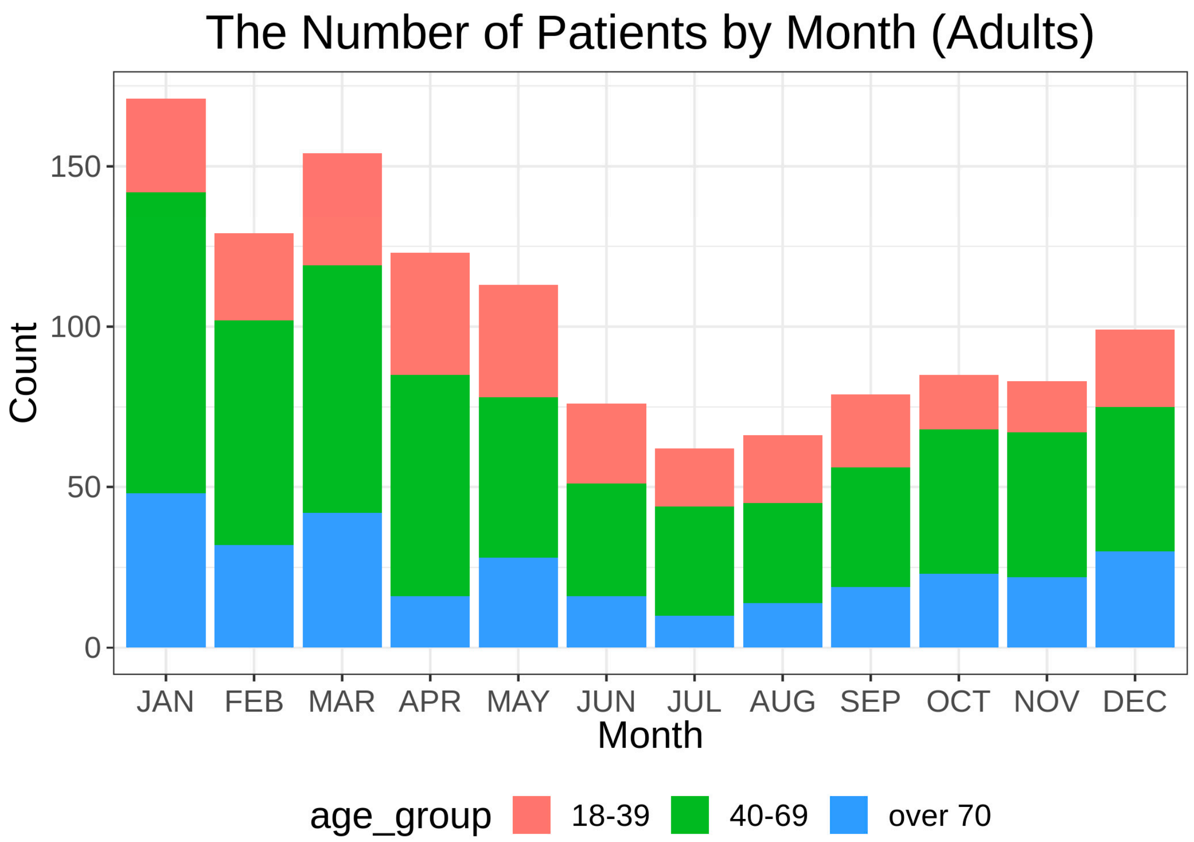

Supplementary figure 2. The number of epistaxis presentation by month of the adults group during 2015-2019. We further divided the age of the adult group in three components, ‘18-39,’ ‘40-69,’ and ‘over 70.’ All age groups in adults show a similar pattern of having smaller numbers of patients in summer periods.
