# Supplementary material for: Particulate Matter 10 (PM10) Is Associated with Epistaxis in Children and Adults
Source: Int J Environ Res Public Health. 2021 Apr 30;18(9):4809. doi: 10.3390/ijerph18094809 (PMC8124263; doi:10.3390/ijerph18094809)
Supplement: Supplementary file 1 [file ijerph-18-04809-s001.zip › Supplementary figure 3.pdf]

Children: Adults:

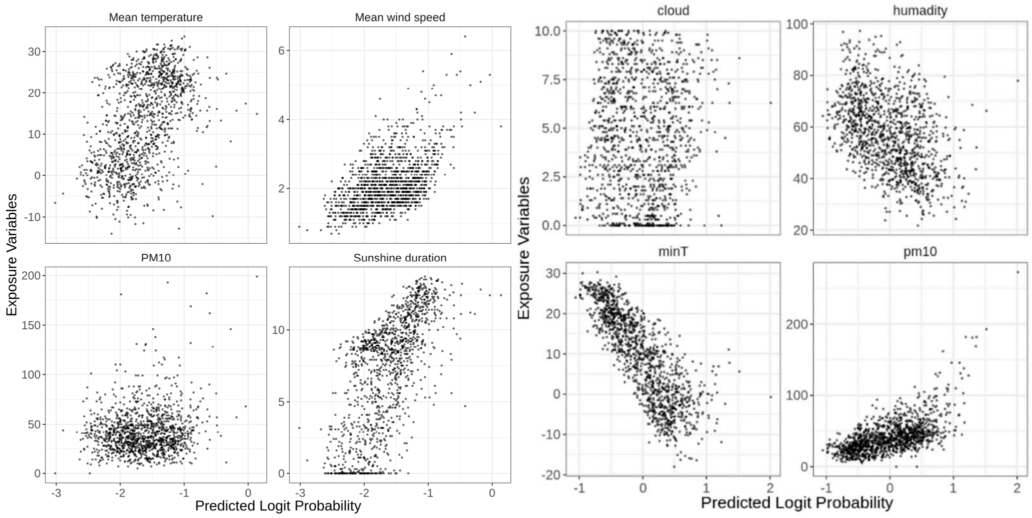

Supplementary Figure 3.
To check the linearity assumption, we visualized the relationship between logit value of the predicted probability and statistically significant exposure variables. Especially for temperature and pm10, we found a strong linear relationship with predicted logit probability
