# Supplementary material for: Particulate Matter 10 (PM10) Is Associated with Epistaxis in Children and Adults
Source: Int J Environ Res Public Health. 2021 Apr 30;18(9):4809. doi: 10.3390/ijerph18094809 (PMC8124263; doi:10.3390/ijerph18094809)
Supplement: Supplementary file 1 [file ijerph-18-04809-s001.zip › Supplementary Table 1.pdf]

Supplementary Table 1. Details of exposure variables.

	No. of patients (or days)	Mean	SD	Range	Lower 95% CI	Upper 95% CI
Age	1557	44.29	30.75	1~94	29.53	31.97
Epistaxis Count	ccc	0.93	1.01	0~6	0.88	0.98
PM10	1704	42.3	27.54	0~627	40.92	43.65
Cloud	1704	4.61	3.04	0~10	4.46	4.76
Humidity	1704	57.14	14.38	21.9~97.3	56.43	57.85
Min. humidity	1704	35.15	14.75	7~91	34.42	35.88
Max Temperature	1704	18.23	11.16	-10.7~39.6	17.68	18.79
Min Temperature	1704	8.86	11.03	-18~30.3	12.59	13.68
Air pressure	1704	13.48	10.97	-14.8~33.7	1005.85	1006.63
Solar Radiation	1704	1006.2	7.89	983.8~1026.8	13.09	13.76
Sunshine Duration	1704	13.43	6.76	0.02~29.2	7.08	7.46

Max wind speed	1704	7.27	3.91	0~13.7	5.20	5.38
Mean wind speed	1704	5.29	1.84	1.6~14.9	2.14	2.21
